# Conversation about Serostatus decreases risk of acquiring HIV: results from a case control study comparing MSM with recent HIV infection and HIV negative controls

**DOI:** 10.1186/1471-2458-14-453

**Published:** 2014-05-14

**Authors:** Claudia Santos-Hövener, Ruth Zimmermann, Claudia Kücherer, Jörg Bätzing-Feigenbaum, Stephan Wildner, Osamah Hamouda, Ulrich Marcus

**Affiliations:** 1 Department for Infectious Disease Epidemiology, HIV/AIDS, STI and Blood-borne Infections Unit, Robert Koch Institute, Berlin, Germany; 2 Department of Infectious Diseases, Unit for HIV and other Retroviruses, Robert Koch Institute, Berlin, Germany; 3 Central Research Institute of Ambulatory Health Care in Germany, Berlin, Germany; 4 INTERLAB central lab services - worldwide GmbH, Munich, Germany

**Keywords:** MSM, KABP, Transmission risk, Serosorting, Recent HIV infection, Case control study

## Abstract

**Background:**

Data on knowledge, attitudes, behaviour and practices (KABP) of persons with recent HIV infection compared to controls with negative HIV test result provide information on current risk patterns and can help to re-focus HIV prevention strategies.

**Methods:**

From March 2008 through May 2010, persons newly diagnosed with HIV (cases) and HIV-negative controls were recruited by physicians in Germany. To distinguish recent (< 5 months) from longstanding (> 5 months) infection, dried blood spots from people newly diagnosed with HIV were tested with the BED IgG-capture ELISA. Cases and controls completed a KABP-questionnaire. We compared cases with recent infection and controls among men having sex with men (MSM) regarding reported risk behaviour in the previous 6 months. To detect differences, unadjusted Odds Ratios (OR) were calculated and multivariate analysis was performed.

**Results:**

Cases and controls did not differ in terms of knowledge on transmission risks, HIV testing frequency, partnership status, or regarding the frequency of any unprotected sex with partners known to be HIV-positive or assumed to be HIV-negative. Cases more often reported a shorter duration of partnership (< 6 months) with a primary partner than controls (OR = 3.9; p = 0.003) and indicated lower rates of condom use outside of primary relationships, with acquaintances (OR = 2.5; p = 0.01), and with persons met online (OR = 4.5; p = 0.04). Unprotected sex with persons of unknown HIV-serostatus was more often indicated by cases than controls (OR = 3.0; p = 0.003). Having a conversation about HIV serostatus before having sex was associated with a lower risk of infection (OR = 0.2; p = 0.01). In multivariate analysis “being always safe” (always using a condom when having sex in different situations outside of a relationship) and talking about serostatus before sex (OR = 0.23; p = 0.004; OR = 0.14; p = 0.014) were negatively associated with HIV- infection.

**Conclusions:**

There were no significant differences regarding knowledge about HIV-transmission risks among cases and controls. Differences in risk behaviour were observed regarding unprotected sex with partners of unknown HIV-serostatus and duration of primary partnership at the time of diagnosis, suggesting some HIV-transmissions occurring in newly formed partnerships. The practice of discussing serostatus with prospective sex partners before engaging in sex seems to be protective for HIV-transmission.

## Background

The HIV epidemic in Germany is a concentrated epidemic with sub-populations, especially men having sex with men (MSM), being disproportionately affected by HIV [1]. Recent estimates indicate that the majority (51,000) of the 78,000 persons living with HIV in Germany are MSM, followed by 17,000 persons with heterosexual transmission and approximately 8,500 people who inject drugs. The estimated HIV incidence in MSM steadily declined after the beginning of the epidemic in the early 1980’s. However, since 2001 HIV incidence among MSM has increased again [2] and this group still remains one of the key populations for HIV infection, transmission and prevention in Germany.

Reasons for the increasing HIV incidence in MSM have been attributed to changes in risk behaviour, such as increasing number of sex partners, higher frequency of unprotected anal sex [2,3] and substance use [4,5]. An increase of reported syphilis cases and other bacterial sexually transmitted infections may also contribute to increasing HIV incidence by increasing the transmission probability for HIV [2,6-10]. In addition, research has shown that MSM have implemented various risk reduction and management strategies, such as choosing a partner based on disclosed or assumed serostatus (serosorting) or selecting sexual activities based on HIV-status (seropositioning) [11-14]. In gay couples practices like “negotiated safety” [15], intending to reduce risks of HIV-acquisition outside of steady relationships while allowing condom-less sex between HIV seroconcordant steady partners are commonly used to reduce risk of HIV-acquisition and transmission within relationships [16]. Whether or not these risk reduction strategies are effective depends on multiple factors such as honest disclosure of serostatus, regular testing for HIV (after potential risks), occurrence of sexually transmitted infections and consistency of behavior [12,13,17].

All these factors show the importance of research on preventive and risk behaviours among MSM to understand the specific prevention needs of this group. Studies on knowledge, attitudes, behaviour and practice (KABP) are needed [18-21] to provide an understanding of risk behaviour and help to identify possible protective factors that can be utilized for prevention messages and intervention planning [22]. Ideally KABP-studies are combined with biological data to determine serostatus of respondents and compare risk and preventive behaviour of individuals with different serostatus [22-24].

UNAIDS and WHO developed the concept of second generation surveillance, combining both the monitoring of biological (new cases of HIV/AIDS) and behavioral indicators (e.g., sexual behaviour, use of protection). This type of surveillance is recommended in particular for populations at high risk to acquire HIV infection, such as MSM [22,25]. Following this concept, HIV surveillance in Germany is based on three pillars: Mandatory reporting of new HIV diagnoses (since 1988), long-term cohort studies focusing on treatment and long-term effects in people living with HIV, as well as studies among sub-populations most at risk for HIV infection. Monitoring of recent HIV infections was first introduced into the German HIV surveillance system in 2005 for assessment of current dynamics of the HIV epidemic in Germany and for identification of populations at risk for HIV-infection. To determine recency of infection (< 5 months) the BED IgG-capture-ELISA is used [26,27].

Combining KABP and biological data can be helpful to get information about risk and protective behaviour in individuals with different serostatus. By adding testing for recency of infection in newly diagnosed cases, it becomes possible to restrict comparative analysis to individuals with recent HIV infection who have been at risk within the previous five months. The objective of this study was the identification of behavioural factors protecting MSM from HIV acquisition.

## Methods

### Study design and data collection

**Figure 1:**
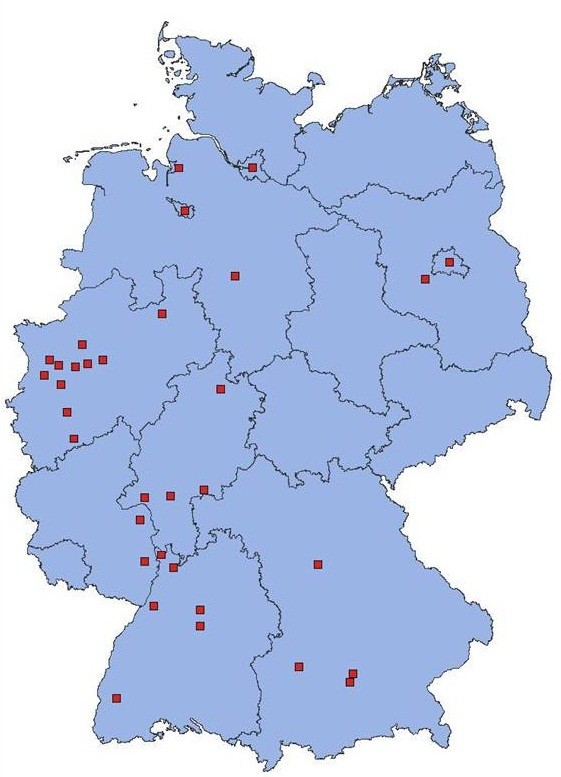
**Cities with recruitment sites (map of Germany with federal states).** We have the copyright to this file.

Participant recruitment took place from March 2008 through May 2010. 72 study sites offering HIV testing in 35 cities from different regions in Germany (Figure 1), accounting for approximately 70% of all reported HIV diagnoses from 2000–2006 were included. Study sites included private practices (74%), outpatient clinics (13%), local health authorities (11%), and other agencies (0.2%).

Participants were recruited through convenience sampling, and participation was offered right before or sometimes after receiving an HIV test result. Eligible persons were informed about the study by test counsellors or physicians, and written informed consent was obtained from each participant.

Although we tried to recruit persons from all sub-groups with higher risk of HIV infection (MSM, migrants, intravenous drug users, persons with heterosexual transmission), sub-samples other than MSM were too small for analysis.

Cases were defined as men who accessed HIV testing services, reported having sex with other men, and being diagnosed with HIV. Controls were male individuals who accessed HIV testing services, reported having sex with other men, and having received a negative test result.

Inclusion criteria for cases and controls were i. age 18 or older. ii. signed informed consent. iii. having received an HIV test result. For cases inclusion criteria was extended to: iv. confirmed HIV-diagnosis within the last 12 weeks (ELISA reactive and Immunoblot positive). Cases and controls were matched for age group, and WHO-region of birth/origin.

Test counsellors or physicians completed a questionnaire for cases and controls (“physicians questionnaire”), covering: i. demographic information, ii. transmission risk, iii. last negative test result, iv. probable source of transmission, v. symptoms, vi. in cases information on anti-retroviral therapy.

When accessing pre-test counselling and before receiving the HIV-test result, cases and controls were asked to complete a standardized questionnaire (“patients’ questionnaire”), containing questions on KABP and other information. The following sections were covered in the patients’ questionnaire:

•Personal and demographic information (e.g., level of education, sexual orientation, partnership status, country of origin)

•HIV testing history (month and year of last test)

•Knowledge about HIV (HIV epidemiology in Germany, transmission risk (“How do you assess the transmission risk in the respective situation?”) Response categories: High risk, intermediate risk, low risk, no risk, don’t know)

•Effects of anti-retroviral therapy (ART) on disease progression and infectiousness

•Sources of information about HIV (“Where did you get information about HIV?”)

•Condom use with different partners during the last six months (at the beginning of a new relationship, with primary partner, outside of primary relationship, sex with an acquaintance, sex with a person met online, anonymous sex, sex with an HIV-positive person). Response categories: always, mostly, rarely, never)

•Reasons for not using condoms at last unprotected intercourse (multiple response options e.g., ”I assumed that my partner was negative.” “We had a face-to-face conversation about serostatus before sex.”)

•Risk behaviour

•Sex during the last 6 months

•Number of sex partners within last 6 months

•Unsafe sex during the last 6 months (vaginal/anal/oral) (with persons you assumed were HIV-negative, with persons with unknown serostatus, with persons with HIV)

•History of sexually transmitted infections (STI) (syphilis, gonorrhoea, chlamydia, herpes, venereal warts)

Physicians’ and patients’ questionnaire, as well as the informed consent form, were pre-tested in a pilot study in Berlin in 2007 [26,27] and adapted afterwards. The patient questionnaire was available in German, English and French. After the first year of data collection, some items were modified: The response category “with person met online” was introduced into the questions on condom use with different partners, and the response option “I was certain that sex partner was HIV negative, because we talked about our serostatus” was added as possible category to the questions on reasons for not using a condom at last sex. The latter was added, because a high number of study participants listed this as a reason for not using condoms in the “other reasons” option.

A unique identifier was assigned to all patients’ questionnaires and it was impossible to trace patients’ identities. Patients participated voluntarily and did not receive compensation. Study sites received an allowance of €30 for every case and €20 for a matched control. Questionnaires were sent to Robert Koch Institute (RKI) and entered into an Access-database. Ethics approval was received from the ethics board at the Charité Berlin (medical university and clinic).

### Laboratory tests

Capillary blood was obtained from cases by finger prick and then applied onto filters (Whatman #903). Filters were dried for at least three hours and sent to the HIV-laboratory at RKI via regular mail. The BED IgG-capture ELISA (BED-CEIA, Calypte Biomedical Corp., Lake Oswego, USA) was performed from dried blood spots (DBS) to distinguish recent (acquired within the last 5 months) from longstanding (> 5 months) infections. The evaluation of the test and validation for its use from DBS is described elsewhere [28,29]. Sensitivity and specificity of BED-CEIA were acceptable (82% and 85% respectively) and comparable to values published earlier [30]. We increased specificity and positive predictive value by using a lower cut-off for the normalized optical density (from 0.80 to 0.65) as described previously [27] and increased specificity and positive predictive values to 97.6% and 93.6% respectively. Consequently, samples classified as “recent” were very likely to truly be recently acquired infections. Results of BED-CEIA were linked with responses from the physicians’ and the patients’ questionnaires.

### Data analysis

Data was analyzed using SPSS 20.0. We compared reported knowledge about HIV, partnership status, duration of partnership, condom use and sexual risk behaviour between recently infected MSM and HIV-negative controls. We created the variable “always safe” which was defined as always using a condom when having sex outside of a relationship, with an acquaintance, with a person met online, a stranger, or with an HIV-positive person or not engaging in these situations. To detect differences between cases and controls chi-square test and unadjusted Odds Ratios (OR) were calculated. A multivariate analysis was subsequently conducted including factors found to differ between cases and controls using a significance level of p ≤ 0.05 of all results in bivariate analyses.

## Results

### Study population

The study population consisted of 105 cases with recent HIV-infection and 105 matched controls.

Mean age of cases and controls was 34 years and the majority of the study population was between 30–44 years of age (52%) or younger (35%) (Table 1). Most common country of origin was Germany (90%), followed by Western European (cases 7%; controls 5%), Central European (5%) or Latin American countries (2%). Level of education was high, with approximately 50% of study population having a high school or university degree (Table 1). There were no significant differences in level of education between cases and controls. 3% of cases and 0% of controls reported intravenous drug use in the previous 6 months.

**Table 1:** Study population: demographic characteristics of cases and controls

	**Cases (n = 105)**	**Controls (n = 105)**
**Age**		
18-29	36%	34%
30-44	52%	51%
> 45	11%	14%
**Level of education**		
Secondary school certificate or completed 8/9th grade	17%	15%
Completed 10th grade	30%	27%
High school graduate	27%	25%
University degree	23%	33%

### Knowledge on HIV and HIV testing

The level of knowledge about HIV as well as on transmission risks was high in the study sample with no difference between cases and controls. The majority of cases (97%) and controls (94%) recognized unprotected receptive anal sex with ejaculation as high risk for HIV transmission. Both groups were likely to overestimate transmission risk; 73% of cases and 57% of controls considered oral sex with ejaculation as high-risk behaviour (Table 2).

**Table 2:** Transmission risk perceptions in different situations (cases and controls)

	**High**	**Medium**	**Low**	**No risk**	**Don’t know**
**Everyday contact**					
Cases	1% (n = 1)	1% (n = 1)	12% (n = 13)	85% (n = 89)	1% (n = 1)
Controls	4% (n = 4)	1% (n = 1)	23% (n = 24)	71% (n = 75)	1% (n = 1)
**Needle sharing**					
Cases	98% (n = 103)	1% (n = 1)	0% (n = 0)	0% (n = 0)	1% (n = 1)
Controls	98% (n = 103)	1% (n = 1)	0% (n = 0)	0% (n = 0)	1% (n = 1)
**Unprotected receptive anal sex with ejaculation**					
Cases	97% (n = 102)	3% (n = 3)	0% (n = 0)	0% (n = 0)	0% (n = 0)
Controls	94% (n = 99)	5% (n = 5)	0% (n = 0)	0% (n = 0)	1% (n = 1)
**Unprotected receptive anal sex without ejaculation**					
Cases	53% (n = 53)	34% (n = 36)	9% (n = 9)	0% (n = 0)	2% (n = 2)
Controls	51% (n = 54)	35% (n = 35)	11% (n = 12)	1% (n = 1)	1% (n = 1)
**Unprotected insertive anal sex**					
Cases	60% (n = 59)	30% (n = 30)	9% (n = 9)	1% (n = 1)	1% (n = 1)
Controls	58% (n = 60)	30% (n = 29)	8% (n = 8)	2% (n = 2)	2% (n = 2)
**Unprotected receptive oral sex without ejaculation**					
Cases	15% (n = 15)	41% (n = 41)	35% (n = 35)	7% (n = 7)	1% (n = 1)
Controls	10% (n = 10)	23% (n = 22)	57% (n = 55)	8% (n = 8)	2% (n = 2)
**Unprotected receptive oral sex with ejaculation**					
Cases	74% (n = 73)	22% (n = 22)	4% (n = 4)	0% (n = 0)	0% (n = 0)
Controls	56% (n = 54)	34% (n = 33)	9% (n = 9)	0% (n = 0)	1% (n = 1)
**Unprotected insertive oral sex**					
Cases	20% (n = 21)	26% (n = 26)	40% (n = 40)	9% (n = 9)	3% (n = 3)
Controls	14% (n = 15)	25% (n = 26)	45% (n = 44)	13% (n = 13)	3% (n = 5)

Only 8% of cases and controls agreed with the statement that an HIV positive person with undetectable viral load cannot transmit HIV sexually, whereas the majority (66% cases and 69% controls) did not concur or did not know (26% vs. 24%). The proportion of MSM testing for HIV at least twice within the last twelve months was similar in cases and controls (85% vs. 81%).

### Relationship status and duration of partnership

Most participants were single (44% vs. 46%), or currently living in an allegedly monogamous same-sex relationship (37% vs. 32%). Controls were more likely to live in a non-monogamous (sex with other people accepted) same-sex relationship (2% vs. 7%) or a steady heterosexual partnership (0% vs. 5%). 50% of cases in comparison to 20% of controls had been in a relationship with a primary partner for less than six months (OR = 3.9; p = 0.03), whereas controls more often reported relationships lasting more than 12 months (OR = 0.25; p = 0.001) (Table 3).

**Table 3:** Relationship status, duration of partnership and number of partners

	**Cases**	**Controls**	**OR**	**p-value**	**Confidence interval (CI)**
**Relationship status**					
**Single**	46% (n = 48)	48% (n = 50)	0.9	0.8	0.54–1.59
**“monogamous” homosexual relationship**	39% (n = 39)	32% (n = 34)	1.23	0.5	0.70–2.18
**Non-monogamous homosexual relationship**	2% (n = 2)	7% (n = 7)	0.3	0.09	0.06–1.34
**“monogamous” heterosexual relationship**	0% (n = 0)	5% (n = 5)	0.5	0.02	0.42–0.56
**Non-monogamous heterosexual relationship**	0% (n = 0)	2% (n = 2)	0.5	0.15	0.43–0.56
**Duration of relationship**					
**< 6 months**	50% (n = 22)	20% (n = 10)	3.9	0.003	1.57–9.70
**6–12 months**	11% (n = 5)	8% (n = 4)	1.4	0.6	0.36–5.75
**> 12 months**	39% (n = 17)	71% (n = 35)	0.25	0.001	0.11–0.60
**Number of sex partners**					
**1 partner**	18% (n = 14)	18% (n = 14)	1	0.95	0.44–2.17
**2–20 partners**	70% (n = 53)	78% (n = 60)	0.7	0.25	0.40–1.26
**> 20 partners**	12% (n = 9)	4% (n = 3)	3.3	0.07	0.86–12.8

### Number of sex partners

94% of cases and 93% of controls indicated that they had sex within the last 6 months. Information on number of male sex partners in that time period was available for 76 cases and 78 controls. There was a significant difference in number of sex partners between cases (mean = 11.8; median = 5) and controls (mean = 6.6; median = 4) (p = 0.03 (t-test)). 18% of cases and controls reported being monogamous over the last six months, whereas 12% of cases and 4% of controls reported more than 20 partners) (p = 0.06, 95%). Most cases (70%) and controls (78%) had between 2 and 20 sex partners (Table 3).

### STIs and unprotected anal intercourse (UAI)

23% of cases and 22% of controls reported an STI within the last 6 months. STIs that occurred among the study population were syphilis (11 cases/5 controls), gonorrhea (4 cases/10 controls), venereal warts (5 cases/6 controls), herpes (4 cases/2 controls), chlamydia infection (3 cases/1 control) and three other STIs (1 case/2 controls).

18% of cases and 11% of controls had engaged in UAI with an HIV positive person, a difference which was not statistically significant. Cases more often had UAI with persons of unknown serostatus than controls (62% vs. 49%; OR = 3.1; p = 0.001). With regards to sexual practices, there was no difference in frequency of insertive UAI between the two groups. However, cases more often reported receptive UAI than controls (43% vs. 19%; RR = 3.3; p = 0.005) (Table 4).

**Table 4:** Condom use and partner characteristics

**Unprotected anal intercourse**					
	**Cases**	**Controls**	**OR**		**p-value**	**CI**
**UAI with person with unknown HIV status**	62% (n = 60)	49% (n = 36)	3.1	0.001	1.56–6.10
Insertive	57% (n = 40)	43% (n = 29)	1.77	0.21	0.71–4.40
Receptive	43% (n = 43)	19% (n = 18)	3.3	0.005	1.40–7.64
**UAI with HIV positive person**	18% (n = 17)	12% (n = 11)	0.6	0.21	0.26–1.35
Inconsistent condom use*					
**Beginning of new relationship**	44% (n = 33)	34% (n = 25)	1.5	0.2	0.79–2.99
**During a relationship**	77% (n = 33)	71% (n = 25)	1.3	0.4	0.66–2.84
**Outside of primary relationship**	42% (n = 43)	30% (n = 27)	2.3	0.012	1.20–4.30
**With strangers**	42% (n = 36)	30% (n = 21)	1.71	0.112	0.88–3.34
**With HIV-positive partner**	26% (n = 14)	31% (n = 14)	0.8	0.61	0.33–1.91
**With acquaintances**	62% (n = 49)	43% (n = 31)	2.2	0.016	1.16–4.24
**With person met online**	55% (n = 17)	22% (n = 8)	4.4	0.005	1.53–12.64
Being always safe**					
	9% (n = 9)	29% (n = 30)	0.23	< 0.000	0.11–0.52

*Never, rarely or mostly used a condom.

**Person always used a condom when having sex outside of a relationship or did not have anal sex in the respective situation.

### Reasons for not using condoms during last unprotected (vaginal/anal) sex

85% of cases and 81% of controls marked at least one reason for not using condoms during last unprotected intercourse. Overall the most common reasons for not using condoms were “thought there was no risk” (n = 55) and “hoped that nothing would happen” (n = 49) (Table 4). Cases more often *assumed* that the partner was HIV negative (OR = 3.6; p = 0.003). Being *certain or convinced* that the HIV serostatus of the partner was associated with lower odds of acquiring HIV (OR = 0.3, p = 0.06). Having a conversation about serostatus with a sex partner before engaging in sex was significantly associated with a lower odds of HIV infection (OR = 0.16; p = 0.001) (Table 5).

**Table 5:** Reasons for not using condoms at last unprotected sex

**Reasons for not using condoms**	**Cases (n = 90)**	**Controls (n = 84)**	**OR**	**p-value**	**CI**
No condom available	14% (n = 13)	7% (n = 6)	2.1	0.1	0.79–5.88
Would have disturbed the mood	13% (n = 12)	17% (n = 14)	0.8	0.5	0.56–2.99
I thought there was no risk	38% (n = 34)	25% (n = 21)	1.8	0.7	0.95–3.49
I hoped that nothing would happen	32% (n = 28)	25% (n = 21)	1.4	0.4	0.28–1.44
Condom caused erection problems	11% (n = 10)	14% (n = 12)	0.75	0.5	0.54–3.27
Assumed partner was negative.	28% (n = 25)	10% (n = 8)	3.6	0.003	1.54–8.33
Convinced partner was negative**	10% (n = 9)	20% (n = 17)	0.3	0.06	0.10–1.52
Talked with partner about serostatus before having sex**	3% (n = 3)	19% (n = 16)	0.2	0.001	0.04–0.52

**Smaller *n* due to modified version of questionnaire.

### Condom use

We compared frequency of condom use in various situations. Fifty-six percent of cases and 66% of controls indicated always using condoms in the beginning of a new relationship, whereas 44% of cases and 34% of controls stated using condoms inconsistently. This difference was not statistically significant. There was also no difference in condom use during relationships, with strangers or an HIV positive partner (Table 3). Cases reported lower rates of condom use outside of primary relationships (42%/30%; OR = 2.3; p = 0.012), with acquaintances (62% vs. 43%; OR = 2.2; p = 0.016), and with persons met online (55%/22%, OR = 4.4; p = 0.005). Controls reported more often than cases to have been “always safe” (29% vs. 9%, OR = 4.6; p = ^< 0.000^) (Table 4).

### Results from multivariate analysis

Variables included in multivariate analysis were age, duration of partnership, number of sex partners, UAI (receptive and insertive), unprotected sex with a person of unknown serostatus, conversation about serostatus, and being “always safe” (Table 6). We used two models, one including the variable “conversation about serostatus” and one without. Two factors were negatively associated with the outcome variable (recent HIV infection): Being “always safe” (OR = 0.23; p = 0.004) and having had a conversation about serostatus before sex (OR = 0.14; p = 0.014).

**Table 6:** Results from multivariate analysis (final model)

**Variable**	**Odds ratio**	**p-value**	**CI**
**Age**	0.98	0.539	0.96–1.07
**Conversation about serostatus before sex**	0.18	0.014	0.05–0.71
**Unprotected sex with person with unknown serostatus**	1.47	0.464	0.24–1.90
**Insertive UAI with person with unknown serostatus**	0.83	0.785	0.32–4.47
**Receptive UAI with person with unknown serostatus**	2.13	0.249	0.59–7.69
“Always safe”**	0.23	0.004	0.08–0.625

**Person always used a condom when having sex s outside of a relationship or did not have anal sex in the respective situation.

## Discussion

The present study examines differences of risk and preventive behavior between MSM with recent HIV infection and MSM with a confirmed negative HIV status in Germany. Cases and controls did not differ with regard to knowledge about HIV, relationship status or the occurrence of STIs. However, we must emphasize that screening for asymptomatic STI for MSM not diagnosed with HIV is rare in Germany [31,32], implying that most STI diagnosed in the study population were probably symptomatic. Duration of primary partnership at the time of diagnosis was shorter for cases compared to controls, suggesting HIV transmission in newly formed partnerships. Also, cases had more sex partners than controls, indicating a correlation between the number of sex partners and the risk of HIV transmission. Cases reported less consistent condom use outside of primary relationships, with acquaintances and persons met online. Explicitly addressing HIV serostatus with sex partners before engaging in sexual activities seemed to protect from contracting HIV.

Knowledge on HIV transmission and testing was high among both cases and controls with no difference between the two groups. Most MSM in this study overestimated potential risks. For example, the majority of participants did not know that sex with an HIV-infected person receiving effective ART puts them at low risk for HIV transmission. However, this statement by the Swiss Federal Commission for Sexual Health had been published in 2008 [33] shortly before this study was conducted and, might have been discussed only among people living with HIV at the beginning of this study.

Overall, we showed that MSM in Germany, as a sub-population with a higher risk of HIV transmission, are generally well informed about risks and facts on HIV. This demonstrates once again that knowledge on HIV by itself does not determine risk behaviour [19,34]. Other KABP-studies among German MSM also indicate good overall knowledge of HIV transmission risks; however the degree of knowledge is influenced by level of education [35] which was rather high in our study and might have introduced selection bias.

Notably, 9% of MSM with recent HIV infections reported being “always safe” within the last six months. This is surprising, because these cases had acquired HIV within the last five months and most have been exposed to risk behaviour. One possible explanation for this discrepancy is that participants do not remember or do not want to report risk events. Also, three cases indicated intravenous drug use during the last 6 months and might have not had sexual risk. Furthermore, the specificity of the BED-CEIA is not 100% and there might have been some misclassifications.

### Conversation about serostatus: “Serotalking”

The practice of serosorting, which is defined as having sex without condoms exclusively or preferentially with partners of concordant HIV status and of using condoms with HIV-discordant or HIV status unknown partners, has become increasingly common among MSM [12,14,36,37]. Research has shown that this practice can increase the risk of acquiring HIV and other STIs [13,14,36,38,39], whereas others have found that this practice might decrease risk of infection [11]. Whether serosorting might or might not work as a risk management strategy is influenced by various factors, such as the prevalence of HIV in the population; the explicitness of communication; the proportion of people living with HIV who are aware of being infected; the incidence of new HIV infection among people previously testing negative for HIV and practicing HIV serosorting, and their position in sexual networks; the willingness to disclose HIV infection to a potential sex partner; the context of serostatus communication; and the proportion of people living with HIV under effective ART [14,37].

Our results suggest that the way serosorting is performed might have an impact on HIV transmission risk. One of the key findings was that having an explicit conversation about HIV serostatus before sexual activity reduces the risk of acquiring HIV. This might be attributable to the mode of serostatus communication: namely a direct and explicit conversation might be protective, whereas other ways of serosorting, such as relying on online profiles, or guessing/assuming HIV status based on appearance, might be much less effective. This interpretation is supported by the finding that “assuming my partner was HIV-negative” was associated with an increased risk of being diagnosed with HIV in bivariate analysis. Direct communication compared to non-verbal perceptions reduces the potential for misinterpretations such as “he must be HIV-positive/HIV-negative because he doesn’t insist on condom use”. MSM diagnosed with HIV rarely report UAI with non-steady partners known to be serodiscordant, but relatively frequently report UAI with HIV serostatus unknown partners.

Further research should investigate the utilization of serotalking by persons with different relationship status and thus examine whether serotalking is more likely used among MSM in steady relationships than by MSM engaging in casual sex with non-primary partners. According to former research the duration of partnership might have an impact on truthfully disclosing risk behaviour and serostatus [15]. Also, further research is needed to determine what information and topics serotalking should entail to become an effective risk management strategy.

Of course, a direct and explicit conversation about serostatus might not always be possible or feasible before having sex, e.g., in anonymous sex venues, and consequently “serotalking” cannot be promoted as the “one and only” risk management strategy. In our study, sex with acquaintances or with partners met online was rather common, and in these situations communication about serostatus might be an appropriate risk reduction strategy. Ideally, such communication should contain not only, but also information on recency of test results and number of potential HIV exposures since the last test [40,41].

### Limitations

There are some limitations to consider when interpreting our results. Our study was conducted in 2008–2010, an era when late antiretroviral treatment initiation was still common in Germany (CD4 threshold for treatment initiation was increased from 200 CD4 cells/μl to 350 CD4 cells/μl in a treatment guideline update in September 2008) [42]. Meanwhile, among MSM close to 90% of men diagnosed with HIV are receiving ART. Most MSM who could effectively transmit HIV today are likely to be unaware of being infected [43]. Serostatus communication as a HIV risk management strategy might therefore be less efficient today compared with the situation a few years ago.

With our sampling strategy we only reached MSM who accessed HIV testing services in the respective study sites. Therefore, our study population might include a high number of persons who have a concrete reason to get tested, namely a previous high risk situation. Also, we did not reach MSM who do not access testing. The KABP-questionnaire was not developed specifically for MSM, but we initially tried to recruit participants from all populations relevant for HIV-transmission in Germany. Consequently, some of the questions might not reflect the language or the specific situation of MSM (e.g., no questions on specific sex venues; substance use; in questions on sex with persons with unknown and positive serostatus, first response choice was vaginal sex). Because we reached predominantly MSM with the study, we changed some items of the questionnaire during the study period, which might have created limitations to comparability. Further, some of the questions had many response options, making the questionnaire more complicated and potentially causing missing answers. We did not include questions on UAI in new partnerships in order to understand what happens in the early stages of a relationship. We also did not add questions on substance use and alcohol consumption (except for intravenous drug use), even though this might affect risk behaviour and condom use. Lastly, the sample is rather small (105 cases and controls) and with a bigger sample size we might have been able to detect and identify other relevant risk or protective factors.

## Conclusion

We observed differences in risk behaviour between MSM with recent HIV-infection and HIV-negative controls regarding unprotected sex with partners of unknown HIV-serostatus, and duration of primary partnership at the time of diagnosis, suggesting some HIV-transmissions occurring in newly formed partnerships. Having a conversation about serostatus with prospective sex partners before engaging in sex was associated with lower odds of HIV-diagnoses.

## Competing interests

The authors declare that they have no competing interests.

## Authors’ contributions

JB and RZ designed and conducted the study and were supported by UM and OH. CK and SL tested samples for recency of infection. Data analysis and interpretation was conducted by CSH and supported by RZ and UM. The manuscript was drafted by CSH and critically revised by RZ, UM, OH, CK, LW and JB. All authors read and approved the final manuscript.

## Pre-publication history

The pre-publication history for this paper can be accessed here:

http://www.biomedcentral.com/1471-2458/14/453/prepub

## References

[B1] ECDC/WHO Euro HIV/AIDS surveillance in Europe 2012 2013 Stockholm: ECDC

[B2] RKI Schätzungen der Prävalenz und Inzidenz von HIV-Infektionen in Deutschland Epidemiologisches Bulletin 2012 47 465 476

[B3] Marcus U Voss L Kollan C Hamouda O HIV incidence increasing in MSM in Germany: factors influencing infection dynamics Euro Surveill 2006 11 9 157 160 17075160

[B4] Reback CJ Fletcher JB Shoptaw S Grella CE Methamphetamine and other substance use trends among street-recruited men who have sex with men, from 2008 to 2011 Drug Alcohol Depend 2013 133 1 262 265 10.1016/j.drugalcdep.2013.06.007 23890490 PMC3888192

[B5] Woolf-King SE Rice TM Truong H-HM Woods WJ Jerome RC Carrico AW Substance use and HIV risk behavior among men who have sex with men: the role of sexual compulsivity J Urban Health 2013 90 5 948 952 10.1007/s11524-013-9820-0 23974946 PMC3795185

[B6] Marcus U Kollan C Bremer V Hamouda O Relation between the HIV and the re-emerging syphilis epidemic among MSM in Germany: an analysis based on anonymous surveillance data Sex Transm Infect 2005 81 6 456 457 10.1136/sti.2005.014555 16326845 PMC1745061

[B7] Bremer V Marcus U Hamouda O Syphilis on the rise again in Germany--results from surveillance data for 2011 Euro Surveill 2012 17 29 1 5 22835467

[B8] Marcus U Bremer V Hamouda O Kramer MH Freiwald M Jessen H Rausch M Reinhardt B Rothaar A Schmidt W Zimmer Y MSM-STD-Sentinel Network Understanding recent increases in the incidence of sexually transmitted infections in men having sex with men: changes in risk behavior from risk avoidance to risk reduction Sex Transm Dis 2006 33 1 11 17 10.1097/01.olq.0000187224.10428.31 16385216

[B9] European Centre for Disease Prevention and Control STI and HIV prevention in men who have sex with men in Europe 2013 Stockholm: ECDC

[B10] Sullivan PS Hamouda O Delpech V Geduld JE Prejean J Semaille C Kaldor J Folch C Op de Coul E Marcus U Hughes G Archibald CP Cazein F McDonald A Casabona J van Sighem A Fenton KA Annecy MSM Epidemiology Study Group Reemergence of the HIV epidemic among men who have sex with men in North America, Western Europe, and Australia, 1996–2005 Ann Epidemiol 2009 19 6 423 431 10.1016/j.annepidem.2009.03.004 19460672

[B11] Cassels SA Menza TW Goodreau SM Golden MR HIV serosorting as a harm reduction strategy: evidence from Seattle, Washington AIDS (London, England) 2009 23 18 2497 2506 10.1097/QAD.0b013e328330ed8a PMC2886722 19834319

[B12] Marcus U Schmidt AJ Hamouda O HIV serosorting among HIV-positive men who have sex with men is associated with increased self-reported incidence of bacterial sexually transmissible infections Sex Health 2011 8 2 184 193 10.1071/SH10053 21592432

[B13] Eaton LA Kalichman SC O'Connell DA Karchner WD A strategy for selecting sexual partners believed to pose little/no risks for HIV: serosorting and its implications for HIV transmission AIDS Care 2009 21 10 1279 1288 10.1080/09540120902803208 20024704 PMC2937200

[B14] Mao L Crawford JM Hospers HJ Prestage GP Grulich AE Kaldor JM Kippax SC ‘Serosorting' in casual anal sex of HIV-negative gay men is noteworthy and is increasing in Sydney, Australia AIDS (London, England) 2006 20 8 1204 1206 10.1097/01.aids.0000226964.17966.75 16691075

[B15] Mitchell JW Aspects of gay male couples’ sexual agreements vary by their relationship length AIDS Care 2014 Psychological and Socio-medical Aspects of AIDS/HIV, doi:10.1080/09540121.2014.882491. (Published online February 2014) 10.1080/09540121.2014.882491 PMC4065218 24512593

[B16] Mitchell JW Characteristics and allowed behaviors of gay male couples’ sexual agreements J Sex Res 2014 51 3 316 328 10.1080/00224499.2012.727915 23514544 PMC4322899

[B17] Mitchell JW HIV-negative and HIV-discordant gay male couples’ use of HIV risk-reduction strategies: differences by partner type and couples’ HIV-status AIDS Behav 2013 17 4 1557 1569 10.1007/s10461-012-0388-6 23247364 PMC4103747

[B18] Dubois-Arber F Jeannin A Spencer B Gervasoni J-P Graz B Elford J Hope V Lert F Ward H Haour-Knipe M Mapping HIV/STI behavioural surveillance in Europe BMC Infect Dis 2010 10 1 290 10.1186/1471-2334-10-290 20920339 PMC2959062

[B19] Cowan SA Haff J HIV and risk behaviour among men who have sex with men in Denmark--the 2006 Sex Life Survey Euro Surveill 2008 13 48 1 6 19040825

[B20] Elford J Jeannin A Spencer B Gervasoni JP van de Laar MJ Dubois-Arber F the HIV and STI Behavioural Surveillance Mapping Group HIV and STI behavioural surveillance among men who have sex with men in Europe Euro Surveill 2009 14 47 1 7 10.2807/ese.14.47.19414-en 19941807

[B21] Johnson WD Diaz RM Flanders WD Goodman M Hill AN Holtgrave D Malow R McClellan WM Behavioral interventions to reduce risk for sexual transmission of HIV among men who have sex with men Cochrane Database Syst Rev 2008 Art. No 3 CD001230 DOI: 10.1002/14651858.CD001230.pub2 18646068 10.1002/14651858.CD001230.pub2

[B22] UNAIDS/WHO Guidelines on surveillance among populations most at risk for HIV 2011 Geneva: WHO

[B23] Ogunnaike-Cooke S Second Generation HIV Surveillance in Canada Surveillance and Risk Assessment Division Centre for Communicable Diseases and Infection Control - Public Health Agency of Canada 2009

[B24] Rehle T Lazzari S Dallabetta G Asamoah-Odei E Second-generation HIV surveillance: better data for decision-making Bull World Health Organ 2004 82 2 121 127 15042234 PMC2585900

[B25] ECDC Technical Report: Mapping of HIV/STI behavioural surveillance in Europe 2009 Stockholm: ECDC

[B26] Bätzing-Feigenbaum J Loschen S Gohlke-Micknis S Hintsche B Rausch M Hillenbrand H Cordes C Poggensee G Kücherer C Hamouda O Implications of and perspectives on HIV surveillance using a serological method to measure recent HIV infections in newly diagnosed individuals: results from a pilot study in Berlin, Germany, in 2005 & 2007 HIV Medicine 2009 10 4 209 218 10.1111/j.1468-1293.2008.00672.x 19207597

[B27] Bätzing-Feigenbaum J Loschen S Gohlke-Micknis S Zimmermann R Kücherer C Hamouda O Piloting second generation HIV surveillance in Berlin, Germany, 2005–2007: Risk profile of recently acquired HIV infections in MSM J AIDS HIV Res 2009 1 1 8 17

[B28] Loschen S Baetzing-Feigenbaum J Poggensee G Cordes C Hintsche B Rausch M Dupke S Gohlke-Micknis S Rodig J Hamouda O Kücherer C Comparison of the human immunodeficiency virus (HIV) type 1-specific immunoglobulin G capture enzyme-linked immunosorbent assay and the avidity index method for identification of recent HIV infections J Clin Microbiol 2008 46 1 341 345 10.1128/JCM.01055-07 17977990 PMC2224246

[B29] Loschen S Strohschein K Wiethaus J Bätzing-Feigenbaum J Kücherer C The use of filter-dried plasma spots for HIV-1 viral load determinations and drug resistance analysis 2009 Berlin: 19th Annual Meeting of the Society for Virology in Leipzig: Robert Koch-Institut 1

[B30] Parekh BS Kennedy MS Dobbs T Pau CP Byers R Green T Hu DJ Vanichseni S Young NL Choopanya K Mastro TD McDougal JS Quantitative detection of increasing HIV type 1 antibodies after seroconversion: a simple assay for detecting recent HIV infection and estimating incidence AIDS Res Hum Retrovir 2002 18 4 295 307 10.1089/088922202753472874 11860677

[B31] Schmidt AJ Marcus U Self-reported history of sexually transmissible infections (STIs) and STI-related utilization of the German health care system by men who have sex with men: data from a large convenience sample BMC Infect Dis 2011 11 132 10.1186/1471-2334-11-132 21592342 PMC3121611

[B32] Schmidt AJ Hickson F Weatherburn P Marcus U Comparison of the performance of STI Screening Services for gay and bisexual men across 40 European cities: results from the European MSM Internet Survey Sex Transm Infect 2013 89 7 575 582 23744961 10.1136/sextrans-2012-050973

[B33] Vernazza P Hirschel B Bernasconi E Flepp M HIV-infizierte Menschen ohne andere STD sind unter wirksamer antiretroviraler Therapie sexuell nicht infektiös Schweizerische Ärztezeitung 2008 89 5 165 169

[B34] Tripathi A Ruutel K Parker RD HIV risk behaviour knowledge, substance use and unprotected sex in men who have sex with men in Tallinn, Estonia Euro Surveill 2009 14 48 1 5 10.2807/ese.14.48.19429-en 20003896

[B35] Bochow MLS Sekuler T Schmidt AJ Schwule Männer und HIV/AIDS: Lebensstile, Sex, Schutz- und Risikoverhalten 2010 AIDS Forum DAH 2011 Berlin: Deutsche AIDS-Hilfe

[B36] Zablotska IB Imrie J Prestage G Crawford J Rawstorne P Grulich A Jin F Kippax S Gay men’s current practice of HIV seroconcordant unprotected anal intercourse: serosorting or seroguessing? AIDS Care 2009 21 4 501 510 10.1080/09540120802270292 19266409

[B37] Velter A Bouyssou-Michel A Arnaud A Semaille C Do men who have sex with men use serosorting with casual partners in France? Results of a nationwide survey (ANRS-EN17-Presse Gay 2004) Euro Surveill 2009 14 47 1 8 10.2807/ese.14.47.19416-en 19941805

[B38] Golden MR Stekler J Hughes JP Wood RW HIV serosorting in men who have sex with men: is it safe? J Acquir Immune Defic Syndr 2008 49 2 212 218 10.1097/QAI.0b013e31818455e8 18769346

[B39] Heymer K-J Wilson DP Available evidence does not support serosorting as an HIV risk reduction strategy AIDS (London, England) 2010 24 6 935 936 10.1097/QAD.0b013e328337b029 20234196

[B40] Rietmeijer CA Lloyd LV McLean C Discussing HIV serostatus with prospective sex partners: a potential HIV prevention strategy among high-risk men who have sex with men Sex Transm Dis 2007 34 4 215 219 10.1097/01.olq.0000233668.45976.a1 17179774

[B41] Crepaz N Marks G Serostatus disclosure, sexual communication and safer sex in HIV-positive men AIDS Care 2003 15 3 379 387 10.1080/0954012031000105432 12745398

[B42] Deutsche und Österreichische AIDS Gesellschaft Antiretrovirale Therapie der HIV-Infektion Deutsche Medizinische Wochenschrift 2009 134 S4 15 19172554 10.1055/s-0028-1123965

[B43] RKI Weiterführende Analysen zur HIV-Inzidenz- und -Prävalenz-schätzung 2012 Epidemiologisches Bulletin 2013 45 457 464

